# Gestational weight gain of multiparas and risk of primary preeclampsia: a retrospective cohort study in Shanghai

**DOI:** 10.1186/s40885-023-00254-5

**Published:** 2023-12-01

**Authors:** Chao Chen, Zhijun Lei, Yaoxi Xiong, Meng Ni, Biwei He, Jing Gao, Panchan Zheng, Xianjing Xie, Chengrong He, Xingyu Yang, Weiwei Cheng

**Affiliations:** 1 Department of obstetrics, School of Medicine, International Peace Maternity and Child Health Hospital, Shanghai Jiao Tong University, Shanghai, 200030 China; 2 Shanghai Key Laboratory of Embryo Original Disease, Shanghai, 200030 China; 3 Department of Cardiology, School of Medicine, Shanghai Tenth People’s Hospital, Tongji University, Shanghai, 200072 China

**Keywords:** Multiparous women, Preeclampsia, Gestational weight gain, Z score, Early-pregnancy BMI

## Abstract

**Background:**

In all studies conducted so far, there was no report about the correlation between excessive gestational weight gain (GWG) and the risk of preeclampsia (PE) in multiparas, especially considering that multiparity is a protective factor for both excessive GWG and PE. Thus, the aim of this retrospective cohort study was to determine whether GWG of multiparas is associated with the increased risk of PE.

**Methods:**

This was a study with 15,541 multiparous women who delivered in a maternity hospital in Shanghai from 2017 to 2021, stratified by early-pregnancy body mass index (BMI) category. Early-pregnancy body weight, height, week-specific and total gestational weight gain as well as records of antenatal care were extracted using electronic medical records, and antenatal weight gain measurements were standardized into gestational age-specific z scores.

**Results:**

Among these 15,541 multiparous women, 534 (3.44%) developed preeclampsia. The odds of preeclampsia increased by 26% with every 1 z score increase in pregnancy weight gain among normal weight women and by 41% among overweight or obese women. For normal weight women, pregnant women with preeclampsia gained more weight than pregnant women without preeclampsia beginning at 25 weeks of gestation, while accelerated weight gain was more obvious in overweight or obese women after 25 weeks of gestation.

**Conclusions:**

In conclusion, excessive GWG in normal weight and overweight or obese multiparas was strongly associated with the increased risk of preeclampsia. In parallel, the appropriate management and control of weight gain, especially in the second and third trimesters, may lower the risk of developing preeclampsia.

**Graphical Abstract:**

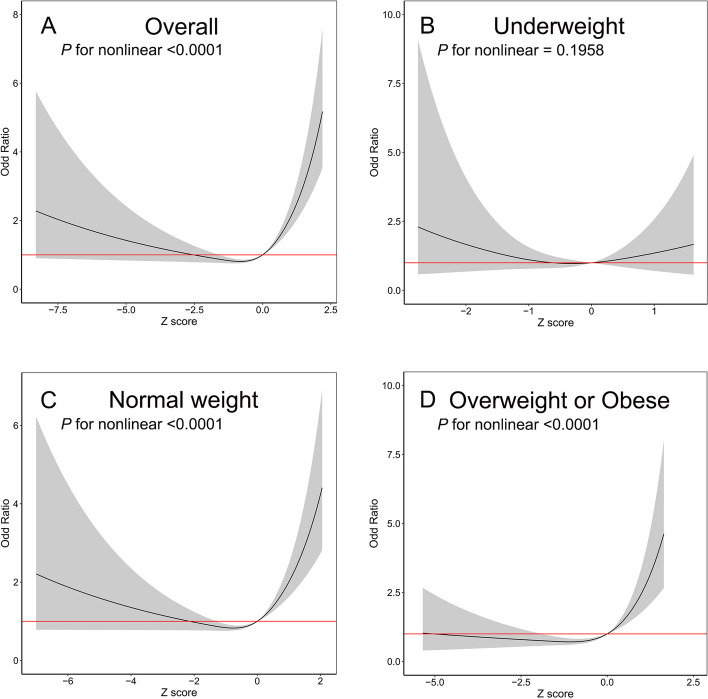

**Supplementary Information:**

The online version contains supplementary material available at 10.1186/s40885-023-00254-5.

## Introduction

Preeclampsia (PE), a pregnancy-associated disease that commonly occurs in pregnant women all over the world, may cause maternal and fetal morbidity and mortality [1]. Apparently, as remarkable impacts on maternal and fetal health, many well-known risk factors, including excessive gestational weight gain (GWG), maternal comorbidities, multiple pregnancy and preexisting hypertension as well as prior preeclampsia, have been identified [2, 3]. Among these risk factors, excessive GWG is a well-established modifiable risk factor for obesity-related adverse pregnancy outcomes, including PE [4–6].

In China, the country introduced the two-child policy in 2016, which has led to a rapid increase in the number of multiparous women [7]. The definition of multiparous women is women who have given birth to one or more children before this pregnancy. Studies have shown that parity could interfere several pregnancy complications, including PE [8–10]. Compared to nulliparous women, in multiparous women, the incidence and severity of PE are significantly lower [11]. Interestingly, GWG of multiparous women is also lower than nulliparous women [8, 12]. These results provoked us whether excessive GWG is also the risk factor of PE in multiparous women.

To more precisely explore the association between these two factors, we additionally excluded pregnant women who had a history of PE, which is a strong predictor of recurrent PE [13], and these women tend to take low-dose of aspirin to prevent PE or control GWG in their subsequent pregnancy, which could be serious confounders of this study [14, 15]. In addition, z score standardized by GWG measurements was used in this study with previously published BMI-specific weight-gain-for-gestational-age charts derived for our population, which ensured that GWG before PE diagnosis can be compared with the GWG without PE at different gestational durations [16, 17].

The aim of this study was to assess the association between GWG in multiparous women and the risk of preeclampsia by different early-pregnancy BMI categories in a large population-based retrospective cohort.

## Material and methods

### Study population selection

**Fig. 1:**
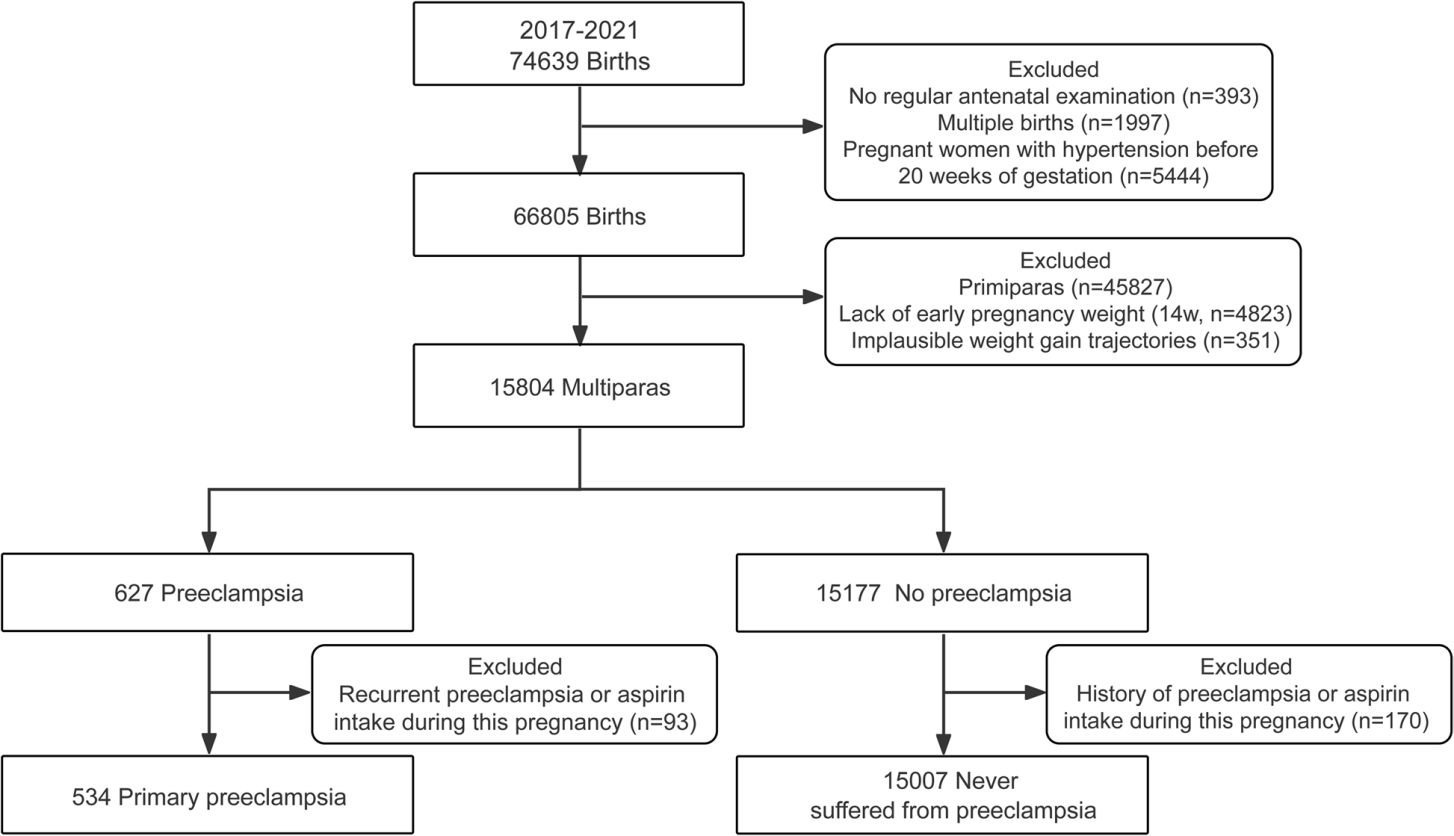
Flow chart of pregnancies with and without preeclampsia in 15,541 multiparous women in Shanghai, January 2017 to December 2021

This was a retrospective and observational cohort study of pregnant women who received their antenatal care and delivered at the International Peace Maternity and Child Health Hospital, Shanghai, China, between January 2017 and December 2021. As shown in Fig. 1, among the 74,639 pregnant women who delivered during this period, a total of 15,541 women with a singleton pregnancy, routine antenatal care, and plausible weight gain records were included for analysis after the following women were excluded: women with no regular antenatal examinations (antenatal care records < 3 times, *n=*393); those with multiple births (*n=*1,997); those with hypertension before 20 weeks of gestation (*n=*5,444); primiparas (*n=*45,827); those missing early pregnancy weight data (before 14 weeks, *n=*4,823); those with implausible weight gain trajectories (*n=*351, an implausible weight gain trajectory was defined by calculating conditional weight percentiles, which were calculated based on a woman’s weight earlier in the pregnancy; women with weight observations that were ≥4 standard deviations from the weight expected based on their weight at the previous visit were excluded); and those with a history of PE or with aspirin intake during this pregnancy (*n=*263).

The present study was approved by the International Peace Maternity and Child Health Hospital Ethics Committee. The need for informed consent was waived by the International Peace Maternity and Child Health Hospital Ethics Committee, as this study was a registry-based study that used anonymized retrospective data. All methods were carried out in accordance with relevant guidelines and regulations.

### Measurements

We defined gestational weight gain as the measured weight (the last measured weight before delivery for patients without PE or the weight at the time of diagnosis for patients with PE) at the time of antenatal care minus the early-pregnancy (<14 weeks) weight. Early-pregnancy BMI was calculated by dividing the weight measured before 14 weeks of gestation (in kilograms) by the square of height (in m^2^), and the pregnant women were divided into underweight (<18.5 kg/m^2^), normal weight (18.5-24.9 kg/m^2^) and overweight or obese (≥25.0 kg/m^2^) groups according to their early-pregnancy BMI. The z score was used for the standardization of GWG, which was converted by previously published BMI-specific pregnancy weight gain-for-gestational age charts for our population and could be used to compare weight gain in different durations of pregnancy [16]. The weight gain z score was subdivided into four groups by quartiles: <25%, 25–50%, 51–75%, and ≥75%.

### Outcome

Preeclampsia was defined as hypertension arising (blood pressure of ≥ 140/90 mmHg) at 20 weeks of gestational age or later with proteinuria (≥++ proteinuria on dipstick test or ≥300 mg/ 24 h) or other signs of end-organ damage (including neurologic complications, pulmonary edema, hematologic complications, acute kidney injury or liver involvement), and primary preeclampsia was defined as suffering from PE for the first time in this pregnancy [18, 19]. PE can be divided into three subtypes according to the difference in occurrence time: early-onset preterm preeclampsia (<34 weeks), late-onset preterm preeclampsia (34-37 weeks) and term preeclampsia (≥37 weeks) [20].

### Statistical analysis

Continuous variables are displayed as the mean (standard deviation SD) or median (interquartile range, IQR) according to their distribution, while categorical variables are shown as a frequency or percentage.

Univariable and multivariable logistic regression analyses were conducted to explore the association between GWG (expressed as a weight gain z score) and PE in multiparous women. The analyses were repeated using the outcomes of early-onset preterm, late-onset preterm, and term preeclampsia. For early preterm (<34 weeks) models, the entire cohort was included in the denominator, while for late preterm and term preeclampsia models, only women who remained pregnant at or beyond 34 and 37 weeks were retained in the denominator, respectively. We also evaluated the linearity of the association between the z score and PE by quartile transformation and restricted cubic spline (RCS) analysis of the z score. The confounders, including maternal age at delivery (years), maternal height (cm), early pregnancy BMI (kg/m^2^), smoking status, education level, mode of conception and gestational diabetes mellitus, were adjusted in multivariable analysis.

To assess the trend of pregnancy weight gain between women with PE and those without PE, a generalized additive mixed model (GAMM) was applied to implement the analysis, which easily accommodates unbalanced, unequally spaced observations [21]. In this study, longitudinal data was the GWG, which was repeated measured when every routinely antenatal care. The time was the interval from early pregnancy before 14 weeks. All models also included random intercept and slope, which allowed each woman’s start of weight gain and the rate of weight gain to vary from the population average. The same confounders mentioned above were also adjusted in the GAMMs.

All tests were two-sided, and a *P* value<0.05 was considered significant. All statistical analyses were completed by R software (version 4.0.5) and SPSS (version 26.0).

## Results

### Study populations

As displayed in Fig. 1, a total of 15,541 multiparous women were enrolled in our study, of whom 534 (3.44%) experienced PE. The baseline characteristics of women with and without PE are detailed in Table 1. Among the women with PE, there were 52 (9.74%), 114 (21.35%) and 368 (68.91%) cases of early-onset preterm, late-onset preterm and term preeclampsia, respectively. Compared with pregnant women in the group that never suffered from PE, those in the group with primary PE had a relatively higher probability of developing gestational diabetes (27.0 versus 19.9%, *P*<0.001), a lower natural conception rate (95.1 versus 96.3%, *P*<0.01) and lower educational attainment (83.1 versus 89.0%, *P*<0.001). In addition, women with PE had a lower fetal weight (3,218.5 versus 3,368.8 g, *P*<0.001) and a relatively higher rate of neonatal hospitalization (15.5 versus 7.9%, *P*<0.001).

**Table 1:** Descriptive characteristics in women with versus without preeclampsia among 15541 parous women in Shanghai, 2017 to 2021

**Characteristic**	**Never suffered from PE** ***n=*** ** 15007**	**Primary PE** ***n=*** **534**	**Primary PE** ***n=*** **534**		
			**Early-onset preterm** **PE ** ***n=*** **52**	**Late-onset preterm PE ** ***n=*** **114**	**Term PE ** ***n=*** **368**
Maternal age, y	34.1 (3.6)	34.3 (4.0)	35.4 (4.2)	34.6 (4.3)	34.1 (3.9)
Gestational age at delivery, week, median (IQR)	38.9 (1.3)	38.6 (1.9)	34.2 (5.8)	37.1 (1.9)	39.0 (1.3)
Maternal height, cm	161.5 (4.8)	161.6 (5.0)	161.6 (5.2)	161.6 (4.9)	161.7 (5.0)
College degree or above (%)	13,361 (89.0)	444 (83.1)^a^	45 (86.5)	88 (77.2)	311 (84.5)
Smoker (%)	40 (0.3)	1 (0.2)	0 (0.0)	1 (0.9)	0 (0.0)
Mode of conception					
Natural conceived (%)	14,449 (96.3)	508 (95.1)^b^	47 (90.4)	106 (93.0)	355 (96.5)
ART (%)	558 (3.7)	26 (4.9)	5 (9.6)	7 (7.0)	13 (3.5)
Gravidity	2.9 (1.1)	3.0 (1.1)	3.2 (1.1)	3.0 (1.2)	3.0 (1.1)
Parity	2.0 (0.2)	2.0 (0.2)	2.0 (0.2)	2.0 (0.2)	2.0 (0.1)
Maternal complications					
GDM (%)	2,992 (19.9)	144 (27.0)^a^	16 (30.8)	36 (31.6)	92 (25.0)
Liver disease (%)	1,158 (7.7)	42 (7.9)	3 (5.8)	6 (5.3)	33 (9.0)
Renal disease (%)	157 (1.0)	9 (1.7)	0 (0.0)	3 (2.6)	6 (1.6)
Thyroid disease (%)	3,742 (24.9)	113 (21.2)	13 (25.0)	24 (21.1)	76 (20.7)
Male sex (%)	7,755 (51.7)	274 (51.3)	26 (50.0)	67 (58.8)	181 (49.2)
Fetal weight, g	3,368.8 (426.5)	3,218.5 (630.1)^a^	2,623.7 (774.1)	3,141.8 (614.6)	3,326.3 (559.8)
Neonatal hospitalization (%)	1,184 (7.9)	83 (15.5)^a^	16 (30.8)	24 (21.1)	43 (11.7)
BMI in early pregnancy, kg/m^2^, median (IQR)	21.8 (3.4)	22.9 (4.4)^a^	22.7 (5.2)	23.3 (4.0)	22.8 (4.2)
Underweight (<18.5) (%)	953 (6.4)	41 (7.7)	5 (9.6)	6 (5.3)	30 (8.2)
Normal weight (18.5-24.9) (%)	11,997 (79.9)	348 (65.2)	31 (59.6)	75 (65.8)	242 (65.8)
Overweight or obese (≥25) (%)	2,057 (13.7)	145 (27.2)	16 (30.8)	33 (28.9)	96 (26.1)
Gestational age at diagnosis of PE, week	...	37.3 (2.8)	30.5 (2.1)	35.7 (0.8)	38.8 (1.0)
Gestational weight gain at delivery, kg, median (IQR)	12.10 (4.90)	12.40 (6.13)	10.60 (5.58)	13.05 (6.95)	12.40 (5.70)
Gestational weight gain at diagnosis of PE, kg	...	10.14 (5.02)	8.28 (3.57)	10.94 (5.28)	10.15 (5.06)
Gestational weight gain at delivery, z score, median (IQR)	-0.27 (1.07)	-0.13 (1.21)^a^	0.03 (1.21)	0.16 (1.54)	-0.24 (1.15)
Gestational weight gain at diagnosis of PE, z score	...	-0.19 (1.14)	0.02 (1.06)	0.00 (1.05)	-0.29 (1.18)

Values are mean (SD) or n (%) unless otherwise stated

*IQR* Indicates interquartile range, *ART* Indicates assisted reproductive technology, *BMI* Indicates body mass index, *GDM* Indicates gestational diabetes mellitus, *PE* Indicates preeclampsia

^a^ *P*<0.001, group no preeclampsia vs group preeclampsia

^b^ *P*<0.01, group no preeclampsia vs group preeclampsia

As expected, women who subsequently developed PE had a higher early-pregnancy BMI (22.9 versus 21.8 kg/m^2^, *P*<0.001) and a relatively higher pregnancy weight gain up to delivery (the corresponding z score comparison was -0.13 vs. -0.27, *P*<0.001) than women who never suffered from PE.

### Association between gestational weight gain z score and preeclampsia in multiparous women

The associations between GWG z score at the time of diagnosis and the risk of PE for multiparous women are shown in Table 2. After adjusting for confounders, we found that the GWG z score was associated with increased odds ratios (ORs) of PE in overall (adjusted OR 1.28, 95% CI 1.15-1.42, *P*<0.001), normal weight (adjusted OR 1.26, 95% CI 1.11-1.43, *P*<0.001) and overweight or obese women (adjusted OR 1.41, 95% CI 1.16-1.74, *P*=0.001), while this association was not significant in underweight women. In addition, the increase in the GWG z score was associated with early-onset preterm and late-onset preterm PE, and the OR for the association between GWG z score and PE was stronger for overweight or obese pregnancies than underweight and normal weight pregnancies in these two subtypes of PE (Supplementary Table 1).

**Table 2:** Association between pregnancy weight gain *Z* score and preeclampsia diagnosis in 15541 multiparas

**BMI category**	**N (case)/N(total)**	**Odd Ratio (95%CI) of a Z Score Increase in Gestational Weight Gain**			
		**Crude**	***P*** ** value**	**Adjusted** ^a^	***P*** ** value**
**Total**	534/15541	1.22 (1.10-1.35)	<0.001	1.28 (1.15-1.42)	<0.001
**Underweight**	41/994	1.06 (0.75-1.51)	0.748	0.94 (0.65-1.39)	0.760
**Normal**	348/12345	1.18 (1.04-1.34)	0.009	1.26 (1.11-1.43)	<0.001
**Overweight or obese**	145/2202	1.36 (1.13-1.65)	0.002	1.41 (1.16-1.74)	0.001

^a^Adjusted for early pregnancy BMI, smoking, maternal height, maternal age, GDM, education level and mode of conception

**Fig. 2:**
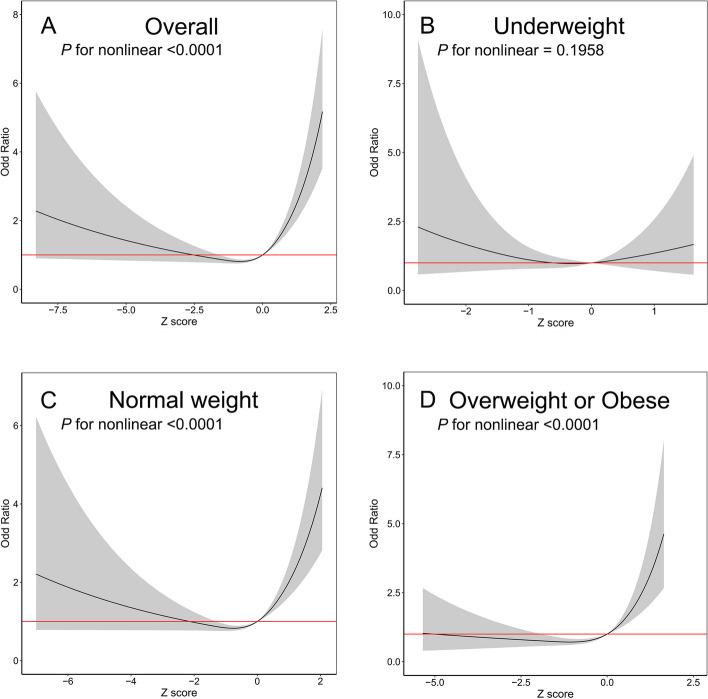
Restricted cubic spline plots of associations between gestational weight gain z score and preeclampsia of multiparous women with 95% confidence intervals by early pregnant body mass index (BMI) in 15,541 multiparous women in shanghai, January 2017 to December 2021. Analyses were adjusted for maternal age at delivery (years), maternal height (cm), early pregnant BMI (kg/m^2^), smoking status, education level, mode of conception and gestational diabetes mellitus

Then, we divided the z score into four categories by quartile to test the linearity of the association between the z score and PE. We found that only the highest quartile of z score was associated with an increased OR of PE in overall, normal weight, overweight or obese women (all *P* for nonlinear<0.001, Supplementary Figure 1). Our RCS analysis further revealed that a J-curve association was identified between z score and PE in overall, normal weight, overweight or obese women (all *P* for nonlinear<0.001, Fig. 2). Naturally, neither pattern was observed in underweight women.

### Gestational weight gain trajectories and preeclampsia risk

**Fig. 3:**
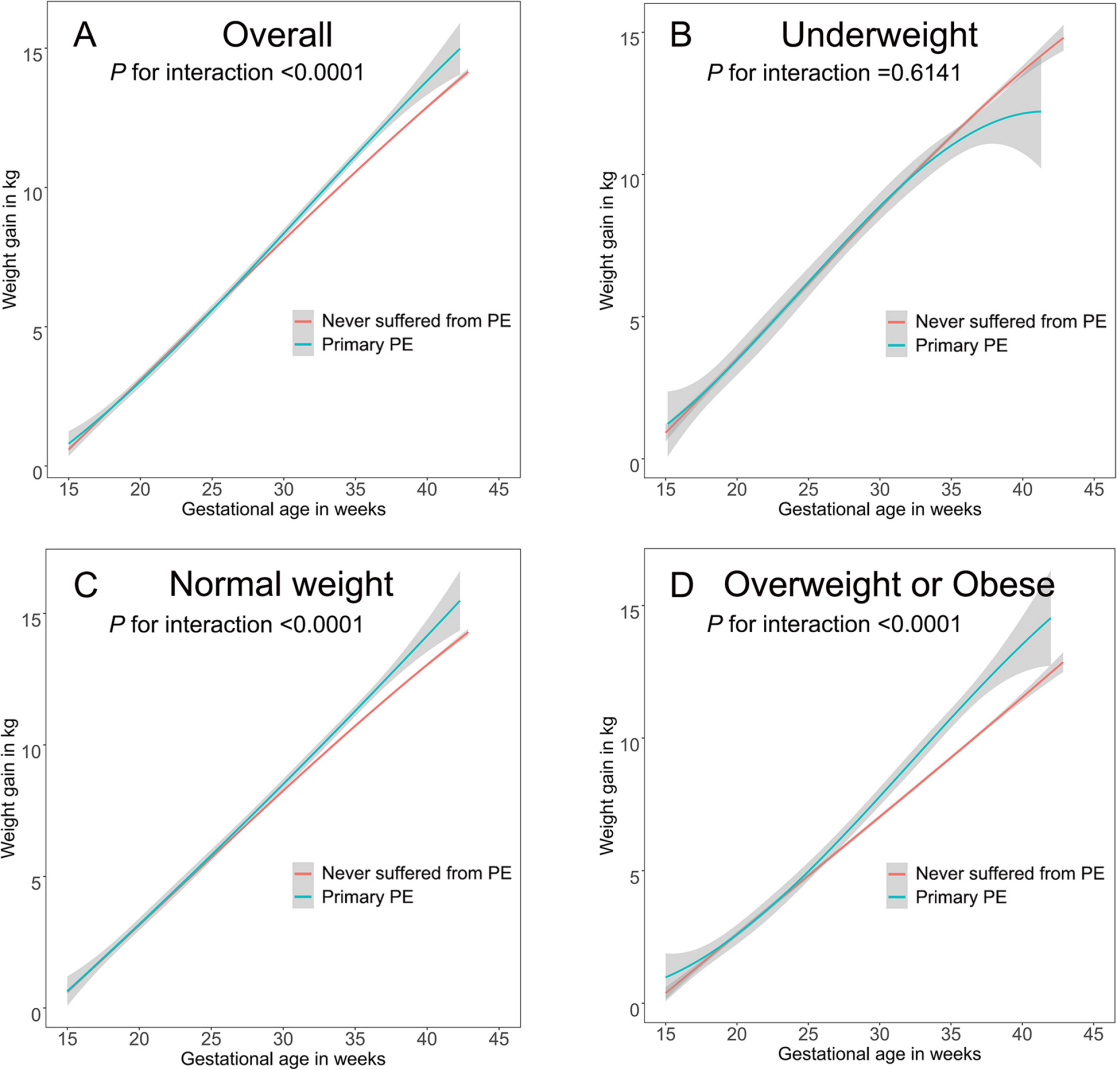
Estimated weight gain (kg) trajectories with 95% confidence intervals of pregnancies with and without preeclampsia by early pregnant body mass index (BMI) in 15,541 multiparous women in Shanghai, January 2017 to December 2021. Trajectories were adjusted for maternal age at delivery (years), maternal height (cm), early pregnant BMI (kg/m^2^), smoking status, education level, mode of conception and gestational diabetes mellitus

We also assessed the trajectories of GWG using a GAMM (Fig. 3). The weight gain patterns were similar for pregnant women with and without PE before 25 weeks of gestation but were totally different after 25 weeks of gestation. For normal weight women, women with PE gained more weight than women without PE, while accelerated weight gain was more obvious in overweight or obese women after 25 weeks of gestation. However, compared with the above two patterns, the GWG patterns were the opposite in underweight pregnant women with PE.

## Discussion

In China, the overall two-child policy has been officially implemented since January 2016, while improving the birth rate, this policy is accompanied by changes in the reproductive structures and pregnancy outcomes of pregnant women [7, 22, 23]. The effects of maternal parity on pregnancies are reflected in many aspects of subsequent pregnancy, such as changes in plasma hormones, placental functions, prenatal and postnatal complications as well as long-term complications [10, 24–27]. Several studies found that the risk of suffering from a series of complications during pregnancy, including PE, was relatively lower for multiparous women than nulliparous women [10, 28, 29]. Multiparas was considered a protective factor in PE, which might be a consequence of a series of changes in cardiovascular adaptations to pregnancy that appear to last at least for a period after delivery, including a large increase in cardiac output and a corresponding decrease in vascular resistance [30]. Similarly, parity also affects the GWG patterns and GWG of multiparas is also lower than those of primiparas [31, 32].

As a well-established modifiable risk factor, many studies have suggested that pregnant women should avoid excessive GWG to reduce the risk of PE [33–35]. Hutcheon JA et al. validated this conclusion in nulliparous of Swedish woman and found that high pregnancy weight gain before diagnosis increased the risk of PE in nulliparous women and was more strongly associated with later-onset preeclampsia than early-onset preeclampsia [34]. Gong X [36] and Zhang S [37] also probed the relationship between GWG of pregnant woman and the risk of PE in China and found that exceed GWG contributed to increased risk of PE. These previously studied populations were either nulliparous women or the whole pregnant women, in contrast, this was the first study to examine the relationship between the GWG of multiparous women and the risk of PE.

In Table 1, compared to pregnancies who never suffered from PE, lower education level, lower nature conceived rate, lower fetal weight, higher prevalence of GDM, higher neonatal hospitalization, higher early pregnancy BMI and higher z score at delivery were observed in PE group. Therefore, the confounders, including early pregnancy BMI (kg/m^2^), education level, mode of conception and GDM, were adjusted in multivariable analysis. As for the higher neonatal hospitalization and lower weight of newborns, we believed that this might be influenced by the mother's preeclampsia, as pregnant women with preeclampsia generally have shorter gestational weeks of delivery and worse prognosis than those with normal delivery.

In this large population-based cohort study, we found that the excessive gestational weight gain of multiparous women was strongly associated with PE in the normal weight group and overweight or obese group. As shown in Fig. 2, the risk of PE significantly increased when z score of multiparas’ GWG was approximately greater than 0, indicating that normal weight and overweight or obese multiparous women with excessive weight gain during pregnancy can increase the risk of PE. However, we could not determine whether there was a correlation between GWG and the risk of PE in underweight multiparous women partially due to the small sample size of the underweight group. The weight gain trajectories of multiparous women who developed PE showed that weight gain began to accelerate at approximately 25 weeks of gestation and constantly diverged until delivery, especially in the overweight or obese group, highlighting the importance of controlling GWG in the second and third trimesters to avoid PE.

However, we recognize that our study has some limitations. First, as the first weight measurement was taken before 14 weeks of gestation, the BMI classification of multiparas was not based on their pre-pregnancy weight. However, the measurement error or memory bias caused by self-measurement of pre-pregnancy weight could hence be avoided. Second, the sample consisted largely of Shanghai women with a relatively high economic status and educational levels, and the generalizability to other women in China is uncertain. This relative homogeneity was also a strength, however, because it reduced the likelihood of confounding factors by differences in patient characteristics. Third, as there are fewer obese women in East Asia than in Western countries, we combined overweight and obese pregnant women to build the models, although the correlation between each of these two groups and the risk of PE may be different. In addition, given the retrospective cohort study design, our analysis would be prone to confounding effects, which we tried to minimize by the logistic regression model. Last but not least, as our study is an observational study, causality between GWG and PE is therefore uncertain and awaits further experimental validation.

## Conclusion

In conclusion, we conducted a large population-based retrospective cohort study in Shanghai to evaluate the association between GWG and the risk of PE in multiparous women and found evidence that when stratified by early-pregnancy BMI, excessive GWG in normal weight and overweight or obese multiparous women was strongly associated with the increased risk of PE. In parallel, the appropriate management and control of GWG, especially in the second and third trimesters, may lower the risk of developing PE.

### Supplementary Information

**Additional file 1: Supplementary Table 1. **Association Between Pregnancy Weight Gain *Z *Score and Three Subtypes of Preeclampsia in 15541 Multiparas.

**Additional file 2: Supplementary Figure 1. **Forest plots of associations between gestational weight gain z score and preeclampsia with 95% confidence intervals stratified by early pregnant body mass index (BMI) in 15541 multiparous women in Shanghai, January 2017 to December 2021. Adjust for maternal age at delivery (years), maternal height (cm), early pregnant BMI (kg/m2), smoking status, education level, mode of conception and gestational diabetes mellitus.

## Data Availability

The datasets generated and/or analyzed during the current study are not publicly available as the additional results from the study are yet to be published; however, they are available from the corresponding author on reasonable request.

## Abbreviations

PE: Preeclampsia
GWG: Gestational weight gain
BMI: Body mass index
RCS: Restricted cubic spline
GAMM: Generalized additive mixed model
OR: Odd ratio
