# Supplementary material for: Gestational weight gain of multiparas and risk of primary preeclampsia: a retrospective cohort study in Shanghai
Source: Clin Hypertens. 2023 Dec 1;29:32. doi: 10.1186/s40885-023-00254-5 (PMC10691081; doi:10.1186/s40885-023-00254-5)
Supplement: Supplementary file 1 — Additional file 1: Supplementary Table 1. Association Between Pregnancy Weight Gain Z Score and Three Subtypes of Preeclampsia in 15541 Multiparas. [file 40885_2023_254_MOESM1_ESM.docx]

**Supplementary Table 1. Association Between Pregnancy Weight Gain *Z* Score and Three Subtypes of Preeclampsia in 15541 Multiparas.**

| **Preeclampsia**  **category** | **BMI category** | **N(case)**  **/N(total)** | **Odd Ratio (95%CI) of a Z Score Increase in Gestational Weight Gain** | | | |
| --- | --- | --- | --- | --- | --- | --- |
|  |  |  | **Crude** | ***P* value** | **Adjusted*** | ***P* value** |
| **Early-onset preterm preeclampsia,**  **<34 week** | **Total** | 52/15541 | 1.66 (1.19-2.35) | 0.004 | 1.84 (1.31-2.62) | < 0.001 |
|  | **Underweight** | 5/994 | 2.04 (0.74-5.49) | 0.174 | 1.65 (0.53-5.27) | 0.392 |
|  | **Normal** | 31/12345 | 1.54 (1.01-2.42) | 0.054 | 1.70 (1.10-2.67) | 0.020 |
|  | **Overweight or obese** | 16/2202 | 1.73 (0.98-3.22) | 0.074 | 2.02 (1.10-3.87) | 0.030 |
| **Late-onset preterm**  **preeclampsia, 34–36 week** | **Total** | 114/15384 | 1.63  (1.30-2.06) | < 0.001 | 1.79 (1.42-2.28) | < 0.001 |
|  | **Underweight** | 6/982 | 0.66 (0.32-1.53) | 0.289 | 0.64  (0.26-1.67) | 0.340 |
|  | **Normal** | 75/12229 | 1.61 (1.21-2.15) | 0.001 | 1.78 (1.33-2.39) | < 0.001 |
|  | **Overweight or obese** | 33/2173 | 2.04 (1.34-3.18) | 0.001 | 2.27 (1.44-3.67) | < 0.001 |
| **Term preeclampsia,**  **≥37 week** | **Total** | 368/14600 | 1.08 (0.96-1.22) | 0.189 | 1.12 (0.99-1.26) | 0.069 |
|  | **Underweight** | 30/928 | 1.06 (0.71-1.62) | 0.767 | 0.95  (0.61-1.51) | 0.824 |
|  | **Normal** | 242/11626 | 1.06 (0.92-1.22) | 0.460 | 1.11 (0.96-1.29) | 0.163 |
|  | **Overweight or obese** | 96/2046 | 1.15 (0.94-1.45) | 0.193 | 1.17 (0.95-1.49) | 0.168 |

*Adjusted for early pregnancy BMI, smoking, maternal height, maternal age, GDM, education level and mode of conception.
