# Supplementary figures and images for: Gestational weight gain of multiparas and risk of primary preeclampsia: a retrospective cohort study in Shanghai
Source: Clin Hypertens. 2023 Dec 1;29:32. doi: 10.1186/s40885-023-00254-5 (PMC10691081; doi:10.1186/s40885-023-00254-5)

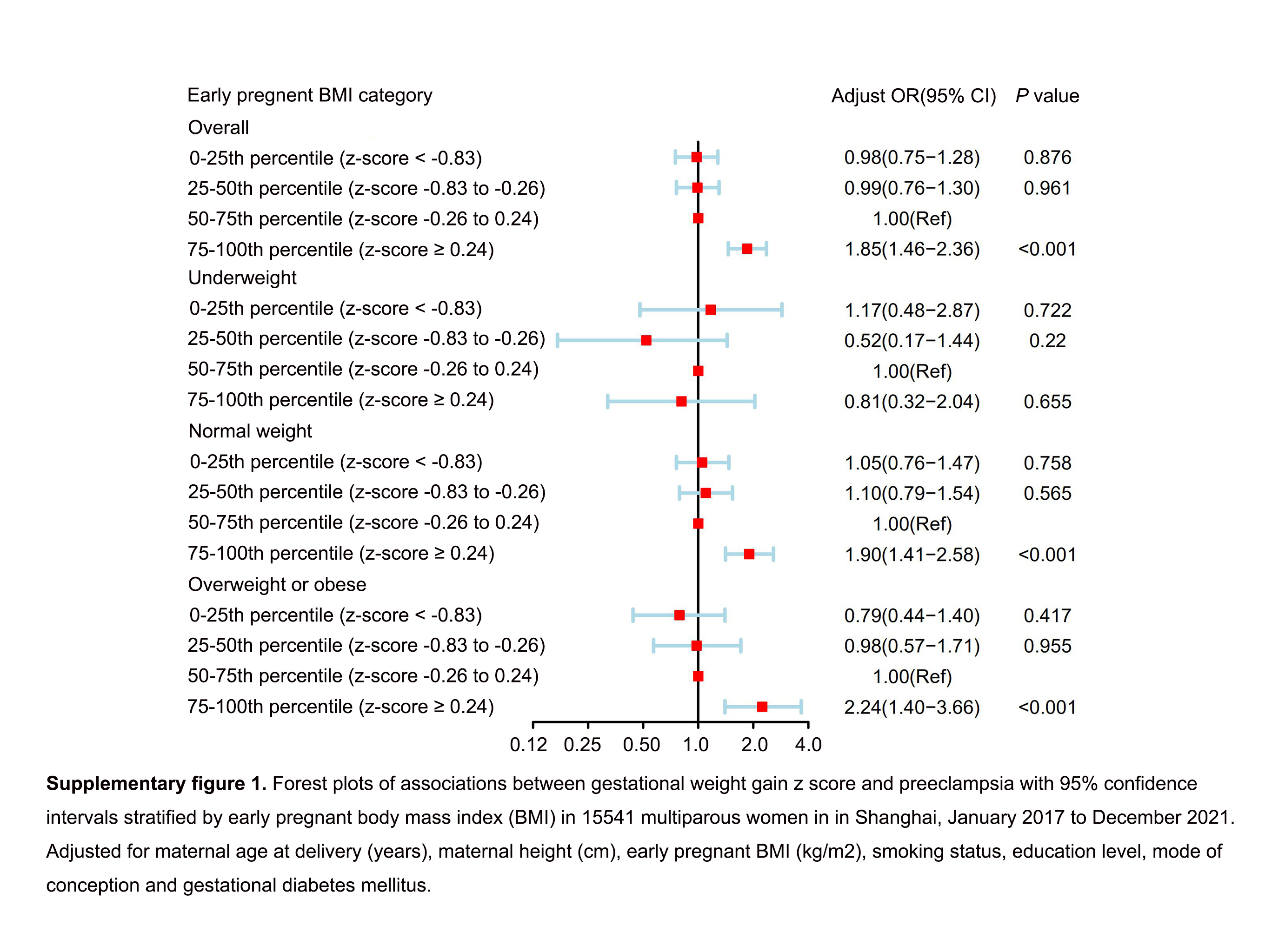

Supplement: Supplementary file 2 — Additional file 2: Supplementary Figure 1. Forest plots of associations between gestational weight gain z score and preeclampsia with 95% confidence intervals stratified by early pregnant body mass index (BMI) in 15541 multiparous women in Shanghai, January 2017 to December 2021. Adjust for maternal age at delivery (years), maternal height (cm), early pregnant BMI (kg/m2), smoking status, education level, mode of conception and gestational diabetes mellitus. [file 40885_2023_254_MOESM2_ESM.jpg]
